# 1-kHz high-frequency spinal cord stimulation alleviates chronic refractory pain after spinal cord injury: a case report

**DOI:** 10.1186/s40981-021-00451-x

**Published:** 2021-06-08

**Authors:** Chiaki Yamada, Aiko Maeda, Katsuyuki Matsushita, Shoko Nakayama, Kazuhiro Shirozu, Ken Yamaura

**Affiliations:** 1 Department of Anesthesiology and Critical Care Medicine, Kyushu University Hospital, Fukuoka, Japan; 2 Operating Rooms, Kyushu University Hospital, 3-1-1 Maedashi Higashi-ku, Fukuoka-city, Fukuoka, 812-8582 Japan; 3 Department of Anesthesiology and Critical Care Medicine, Kyushu University Graduate School of Medicine, Fukuoka, Japan

**Keywords:** Spinal cord injury, Spinal cord stimulation, High-dose stimulation, High-frequency SCS, kilohertz SCS

## Abstract

**Background:**

Patients with spinal cord injury (SCI) frequently complain of intractable pain that is resistant to conservative treatments. Here, we report the successful application of 1-kHz high-frequency spinal cord stimulation (SCS) in a patient with refractory neuropathic pain secondary to SCI.

**Case presentation:**

A 69-year-old male diagnosed with SCI (C4 American Spinal Injury Association Impairment Scale A) presented with severe at-level bilateral upper extremity neuropathic pain. Temporary improvement in his symptoms with a nerve block implied peripheral component involvement. The patient received SCS, and though the tip of the leads could not reach the cervical vertebrae, a 1-kHz frequency stimulus relieved the intractable pain.

**Conclusions:**

SCI-related symptoms may include peripheral components; SCS may have a considerable effect on intractable pain. Even when the SCS electrode lead cannot be positioned in the target area, 1-kHz high-frequency SCS may still produce positive effects.

## Background

Spinal cord injury (SCI) results in partial or complete interruption of transmission from the upper central nervous system to the periphery, resulting in paralysis due to descending motor tract disruption. SCI may additionally induce sensory disorders, spasticity, autonomic dysreflexia, loss of bladder and bowel function, and refractory pain. These symptoms often exert a significant negative lifelong impact after the injury. More than two-thirds of patients with SCI experience chronic pain, with nearly one-third experiencing severe pain [1, 2]. The management of severe neuropathic pain—which is often resistant to conservative treatments and has poorly understood underlying mechanisms—remains challenging [2, 3].

Spinal cord stimulation (SCS) is used to manage chronic neuropathic pain refractory to conservative therapy [4]. Although previous reports have suggested only a mild-to-moderate effect of tonic SCS for SCI [5], there is little information regarding whether the pain that had been effectively treated with SCS is associated with peripheral nerves, central nerves, or both. Additionally, new methods for increased charge delivery, such as sub-perception high-frequency, and burst stimulation, may noticeably reduce refractory neuropathic pain. Here, we report the successful application of 1-kHz high-frequency SCS in a patient with refractory neuropathic pain secondary to SCI.

This article adheres to the applicable guidelines on Enhancing the Quality and Transparency of Health Research. Written informed consent was obtained from the patient for publication of the case report.

## Case presentation

A 69-year-old male patient was referred to our pain management clinic with a chief complaint of severe pain in both upper extremities. When he was 49 years old, he had a severe traffic accident and was rushed to the hospital. After examination, he was diagnosed with cervical ossification of the posterior longitudinal ligament, for which he had no relevant medical history. He underwent a C3–C6 laminoplasty immediately post-injury. However, his complete bilateral lower extremity paralysis and incomplete upper limb paralysis, as well as his severe upper limb pain symptoms persisted and he was diagnosed with C4 SCI (American Spinal Injury Association Impairment Scale A) [6]. He was prescribed various medications including antiepileptics, antidepressants, and weak opioids, those analgesics did not sufficiently relieve his pain. He underwent rehabilitation once a week to prevent joint contractures.

**Fig. 1:**
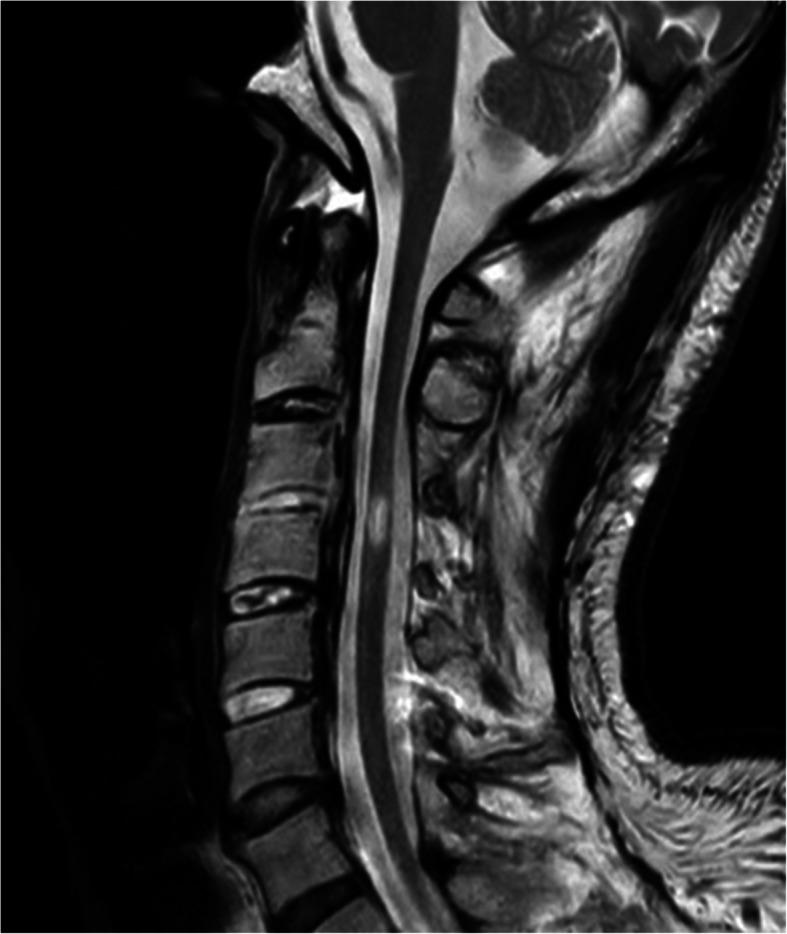
A cervical spine sagittal T2-weighted magnetic resonance image showing a C3-C4 spinal cord lesion

**Fig. 2:**
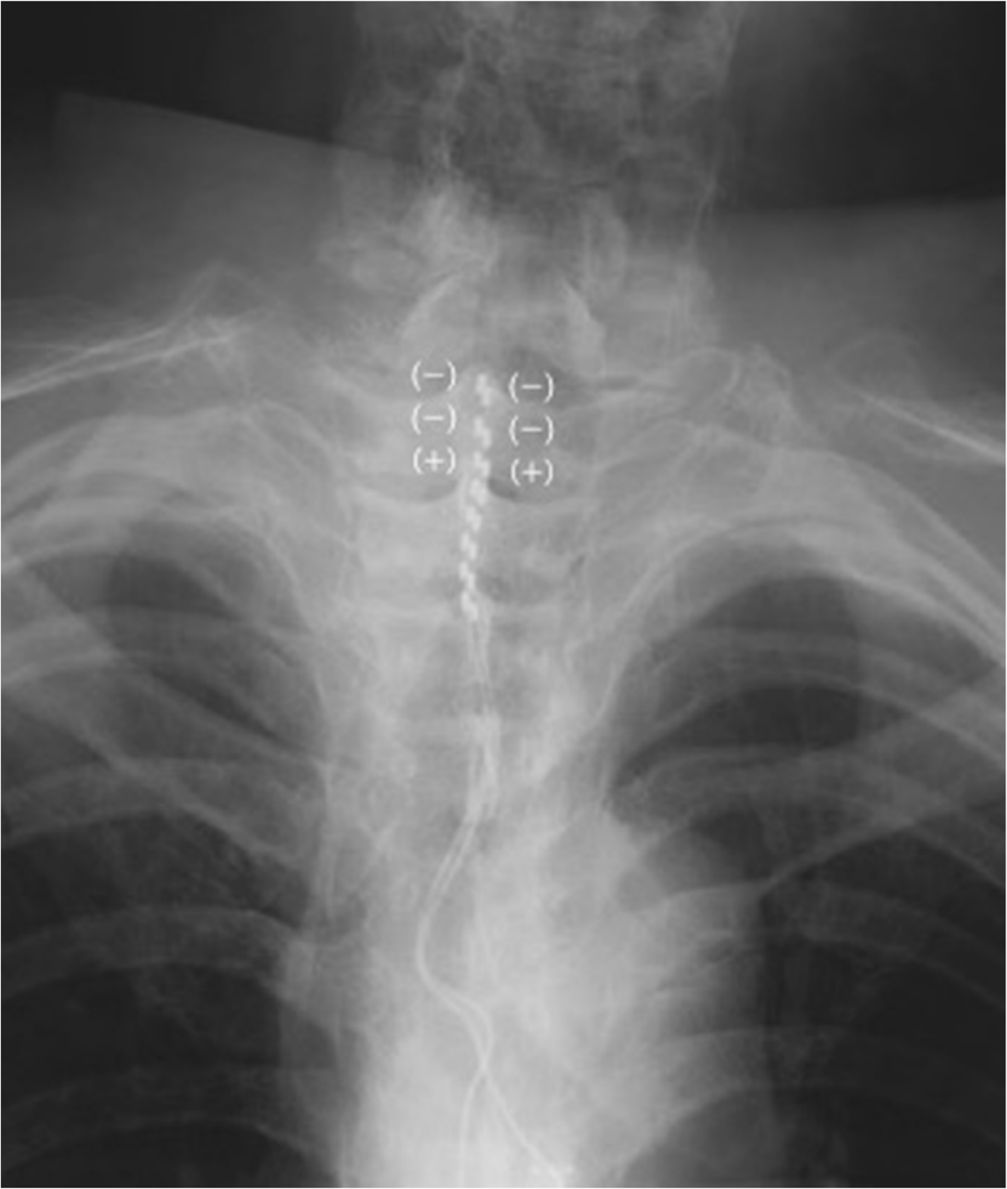
Fluoroscopy image of the spinal cord stimulator. Two 8-electrode epidural leads in the epidural space were positioned at T1-T3. The electrical stimulations of both leads were applied to the first and second (negative) and third (positive) electrodes

**Fig. 3:**
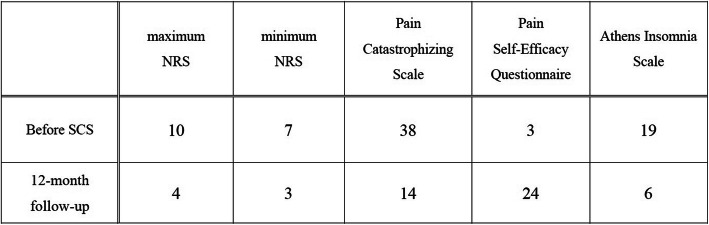
The patient’s pain and sleep progress pre- and post-SCS implantation. The patient’s subjective numerical values concerning all of the questionnaire surveys (NRS, Pain Catastrophizing Scale, Pain Self-Efficacy Questionnaire, Athens Insomnia Scale) improved after SCS implantation

On presentation to our institution, his spontaneous, sharp electrical intermittent pain lasted between several minutes to half an hour and recurred in both upper extremities multiple times daily. The pain severely interrupted the patient’s sleep and quality of life (QOL), with a maximum pain score of 10 and a minimum of 7 on the numerical rating scale (NRS; 0–10). Although he maintained spontaneous breathing, he required maximum assistance with activities of daily living such as feeding and cleaning himself. He had paresthesia in C5–Th3 and sensory paralysis below T4. Manual muscle testing of the bilateral biceps and deltoid muscles revealed scores of 2–3/5, and his lower extremities showed complete paralysis. Muscle atrophy and edematous change were observed in his bilateral upper extremities. Magnetic resonance imaging (MRI) of the cervical spinal cord revealed a high-intensity lesion in the C3–C4 spinal cord (Fig. 1). Although a T1 transforaminal epidural block reduced his pain to NRS 4, the effect was temporary. Therefore, his pain was considered an at-level SCI neuropathic pain, entailing both peripheral and central neuropathic pain. This patient was deemed a good candidate for SCS; therefore, we opted to perform an SCS surgical trial and implantation, and two 8-electrode epidural trial leads (Vectris™ SureScan™ MRI percutaneous leads, Medtronic Inc., Minneapolis, MN, USA) were implanted. Epidural access was obtained at the T6/7 interlaminar space. We had planned to position the tip of the leads at the target cervical dorsal column to obtain paresthesia in the painful region; however, the lead tips were placed at T1 because of epidural space adhesions at the cervical level (Fig. 2). We tried sub-perception SCS stimulation (1-kHz frequency, 90-μs pulse width) as a trial stimulation for 7 days, and the patient experienced > 50% pain improvement (NRS range, 3–5). Hence, he underwent permanent implantation of a pulse generator (Intellis, Medtronic Inc., Minneapolis, MN, USA) in a subcutaneous pocket in the left abdominal region. One month after implantation, the patient’s pain intensity further improved to an average NRS of 3. His sleep and QOL significantly improved. At a 12-month follow-up, the patient did not report any increase in pain. Figure 3 shows the patient’s progress in terms of pain scores and sleep quality.

## Discussion

This article presents two considerations. First, since SCI-related symptoms may include a peripheral component, SCS may help improve intractable pain. Second, sub-perception SCS using 1-kHz stimuli may ameliorate neuropathic pain, even if the SCS electrode cannot be placed in the appropriate space.

According to the International Spinal Cord Injury Pain Basic Data Set classification, SCI-related neuropathic pain can imply an at-level or below-level neurological level of injury (NLI) [2, 3]. At-level neuropathic pain is defined as pain within a region spanning one dermatome rostral and three dermatomes caudal to the NLI, while below-level refers to neuropathic pain located more than three dermatomes below the NLI. Although it remains unclear whether at-level and below-level pain share identical mechanisms or have different etiologies, it is conceivable that at-level pain results from damage to roots and nerves at or around the injury level [1] while below-level pain has been hypothesized to be induced by interruption of ascending sensory tracts or development of phantom pain in deafferented regions. Patients with at-level pain experience pain in response to tactile stimuli about twice as frequently as patients with below-level pain [7]. Thus, peripheral nerves may be more involved in at-level, rather than below-level, pain. Although we do not know to what extent at- or below-level SCI pain is associated with peripheral nerves, it is possible to determine whether the SCI pain includes peripheral components or not using peripheral nerve blocks. In our patient, the severe pain in the bilateral upper extremities—approximately three dermatomes below the C4 NLI—was highly suggestive of at-level neuropathic pain. The temporary symptom improvement conveyed through the use of an epidural block implied a peripheral component. Although SCI-related pain is considered as central neuropathic pain, our patient’s symptoms may have resulted from a combination of factors, including peripheral nerve involvement.

SCS is a low-risk and cost-effective treatment that produces significant neuropathic pain relief, especially in patients with post-lumbar surgery syndrome (PLSS) and complex regional pain syndrome [8, 9]. Published studies and evidence-based guidelines recommend SCS for peripheral neuropathic pain and ischemic pain rather than central pain. Moreover, patients with SCI might respond to SCS for pain at the injury level as opposed to diffuse pain below the level of injury [10].

SCS improves pain through modifying the stimulation parameters (i.e., electrode position, frequency, amplitude, and pulse width). Tonic SCS has been achieved with about 40- to 80-Hz frequency and a range of 200- to 500-μs pulse width for pain relief and comfortable paresthesia, applied in accordance with gate control theory [11]. Tonic SCS delivers mild electrical pulses and stimulates Aβ fibers in the dorsal column, subsequently producing an analgesic effect via two routes. Antidromic stimulation of Aβ fibers leads to the modulation of GABAergic interneurons in the dorsal horn, whereas orthodromic stimulation of Aβ fibers activates the descending pain modulation system (DPMS) in the supraspinal areas. However, despite several mechanisms, in tonic SCS, it is considered that paresthesia overlap in the painful area is essential for ameliorating pain symptoms [11]. Recently, several programming approaches, known as sub-perception or high-dose SCS, to increase charge delivery involving kHz frequency and burst have been proposed to provide further pain control. These programs may significantly alleviate neuropathic pain without paresthesia overlap [12, 13]. Several clinical studies have reported that 1-kHz high-frequency SCS for patients with chronic back and leg pain provided improvement in pain relief, QOL, and patient satisfaction [14–16]. A study using functional MRI in patients with PLSS indicated that high-dose SCS may modulate the brain and brainstem regions of the DPMS [17]. Moreover, in a study using functional MRI, De Groote et al. reported that 10-kHz high-frequency SCS affected pain awareness through involving the dorsolateral prefrontal cortex and the right anterior insula [18]. When a supraspinal effect is one of the important mechanisms of sub-perception SCS, it may be more effective for relieving SCI pain than tonic SCS because many patients with SCI have epidural adhesions due to a past history of spinal surgery. No preclinical or clinical study has examined the efficacy of sub-perception SCS on SCI pain. Therefore, its utility and indications require further investigation using randomized controlled trials.

In conclusion, 1-kHz high-frequency SCS relieved SCI-related chronic refractory pain of our patient. When SCI-related symptoms include a peripheral component, SCS may have a notable effect on intractable pain. Furthermore, even in cases where the SCS electrode leads cannot reach the target area, the use of sub-perception SCS is advisable and should be considered.

## Data Availability

Not applicable

## Abbreviations

DPMS: Descending pain modulation system
MRI: Magnetic resonance imaging
NLI: Neurological level of injury
NRS: Numerical rating scale
PLSS: Post-lumbar surgery syndrome
SCI: Spinal cord injury
SCS: Spinal cord stimulation
